# Community mental health services in Europe: the state of art

**DOI:** 10.1192/j.eurpsy.2024.42

**Published:** 2024-08-27

**Authors:** M. Rojnic Kuzman

**Affiliations:** Zagreb University Hospital Centre and the Zagreb School of Medicine, Zagreb, Croatia

## Abstract

**Abstract:**

In Europe there is significant variability of attitudes, procedure and strategies in clinical care between psychiatrists and settings across different regions and countries. However, there is a significant overrepresentation of data from mental health services from Western and Northern European countries, due a lack of data from Eastern and Central European countries as it has been suggested the Eastern and Central European regions are a “blind spot on the global mental health map”. In respect to community mental health services, Northern and Western countries introduced a large array of multidisciplinary community-based services for people with mental health problems and reorganized the mental health care services towards the community mental health care, replacing largely large hospitals and hospital-based care following recovery-oriented care models with introduction of numerous services which supported full recovery, including supported employment and housing. This process is only in the beginning in the majority of countries in the South and East of Europe. Here we present the data from these countries including the results of the RECOVER-E study (Large-scale implementation of community based mental health care for people with severe and enduring mental ill health in Europe), which incorporated the implementation of community mental health services in five South-eastern European countries.

**Disclosure of Interest:**

None Declared

